# Consumption of a High Quantity and a Wide Variety of Vegetables Are Predicted by Different Food Choice Motives in Older Adults from France, Italy and the UK

**DOI:** 10.3390/nu9090923

**Published:** 2017-08-23

**Authors:** Katherine M. Appleton, Caterina Dinnella, Sara Spinelli, David Morizet, Laure Saulais, Ann Hemingway, Erminio Monteleone, Laurence Depezay, Federico J. A. Perez-Cueto, Heather Hartwell

**Affiliations:** 1 Research Centre for Behaviour Change, Department of Psychology, Faculty of Science and Technology, Bournemouth University, Poole BH12 5BB, UK; 2 Dipartimento di Gestione Sistemi Agrari, Alimentari e Forestali, Universita degli Studi di Firenze, 50144 Florence, Italy; caterina.dinnella@unifi.it (C.D.); sara.spinelli@unifi.it (S.S.); erminio.monteleone@unifi.it (E.M.); 3 Food and Behaviours Department, Bonduelle, 59653 Villeneuve D’Ascq, France; david.morizet@bonduelle.com (D.M.); laurence.depezay@bonduelle.com (L.D.); 4 Centre de Recherche, Institut Paul Bocuse, 69131 Ecully, France; laure.saulais@institutpaulbocuse.com; 5 Faculty of Health and Social Sciences, Bournemouth University, Poole BH12 5BB, UK; aheming@bournemouth.ac.uk; 6 Department of Food Science, University of Copenhagen, Copenhagen 1958 Frederiksberg C, Denmark; apce@food.ku.dk; 7 Faculty of Management, Bournemouth University, Poole BH12 5BB, UK; hhartwell@bournemouth.ac.uk

**Keywords:** vegetable consumption, older people, quantity, variety, food choice, liking

## Abstract

Background: Consumption of a high quantity and wide variety of vegetables is currently recommended for health. Dietary variety can be low, however, particularly for older adults. This study investigated the affective factors associated with the quantity and variety of vegetables consumed by older adults in France, Italy and the UK. Methods: Adults aged 65 years plus completed questionnaires on self-reported vegetable intake (quantity and variety), liking for vegetables, attitudes towards intake, and demographic variables. Results: In 497 older adults (France, *n* = 187, Italy, *n* = 152, UK, *n* = 158), higher quantities of vegetables consumed were associated with a higher age, affluence score and liking for vegetables, and a lower importance in consumption of familiarity (smallest β = 0.11, *p* = 0.03). Greater variety was associated with a higher liking and importance of health benefits, and a lower importance of familiarity (smallest β = −0.11, *p* < 0.01). Higher quantity and variety combined (quantity × variety) was associated with a higher age, liking and importance of health benefits, and a lower importance of familiarity (smallest β = 0.14, *p* = 0.02). Country-specific effects were also found (smallest β = 0.20, *p* < 0.01). Conclusions: These findings demonstrate a role for liking and a lower concern for eating familiar foods in vegetable consumption, and a particular role for concern for health benefits in the consumption of a greater variety of vegetables.

## 1. Introduction

Vegetable consumption is fundamental to good health. A high vegetable consumption has been associated with reduced risk of a number of non-communicable health conditions of major societal impact including cardiovascular disease [1,2], stroke [1,3], type 2 diabetes [4,5], some cancers [1,3,6,7,8], and dementia and cognitive decline [9,10]. Vegetables, furthermore, are a heterogeneous food group, and different vegetables are known to provide different micronutrients of benefit to health [11,12,13]. High intakes of dark green leafy vegetables have been associated with reduced risk for type 2 diabetes [14,15,16], reduced risk for a number of cancers [6,7,17], and with reduced depression [18]. High intakes of cruciferous vegetables, of β-carotene-rich vegetables, yellow- and red-pigmented vegetables, and of fruiting vegetables have been associated with reduced risk from various cancers [6,7,8,17,19,20,21,22], and root vegetable consumption has been associated with reduced type 2 diabetes risk [5]. For a range of health benefits, consumption of an adequate quantity and wide variety of vegetables on a regular basis is recommended [23,24].

Consumption of an adequate quantity and wide variety of vegetables on a regular basis, however, in adult populations across Europe and the US is low [25,26]. While World Health Organization guidelines recommend consumption of at least 160–240 g or 2–3 portions of vegetables/day [23,24], current population records estimate consumption of 74 g (Sweden)–383 g (Romania) vegetables/day across Europe with a mean of 160 g/day, and an additional 6 g (Austria)–41 g (France) and mean 14 g legumes, nuts and seeds/day [25].

Low vegetable consumption in adults has been associated with a number of individual factors. Low consumption is more common in persons of a lower education [27,28,29,30], a lower income [31,32], and a lower social-economic status [27,31,33]. More specific to each individual, high vegetable consumption has been associated with greater liking for the taste of vegetables [34,35,36], greater nutritional knowledge [37,38] and a greater appreciation of health and the value of a healthy diet [37]. High vegetable consumption has also been associated with greater availability of vegetables [35,36], greater familiarity and experience of vegetables [36,38], greater culinary knowledge and culinary confidence [30,34,37,38,39], and with several eating practices including usual consumption of meals as opposed to snacks [29,36], increased time and willingness to prepare and cook home-made meals [35,38,40], and the serving of courses within a meal, vegetable-only courses and larger portions of vegetables [41].

Many of these factors impact on the quantity and variety of vegetables consumed, but in many studies, quantity and variety of vegetables consumed are considered simultaneously or the importance of variety is not considered. Given the differing health benefits from differing vegetables, variety in vegetable consumption is important [11,12,13], and for some individuals, dietary variety can be low.

Older individuals (aged 65 years plus) as a population group can have low dietary variety, or can report an unwillingness to consume certain foods, largely as a result of changes in sensory and gastro-intestinal abilities, increasing disabilities, increased medical conditions and medication usage, and reduced social and economic circumstances [30,33,34,42,43,44,45,46,47]. These factors can limit food shopping, preparation and consumption [30,33,34,42,43,44,45,46,47], and due to their perishable and bulky nature and their fibrous texture and need for cooking, vegetable consumption may be particularly susceptible to these changes in abilities and circumstances [30,33,34,42,43,44,45,46,47].

While abilities and circumstances may deteriorate with age, many of the affective factors associated with vegetable consumption mentioned for adults, such as liking and attitudes, are likely to remain with age and may still apply. Studies considering older peoples’ liking and attitudes towards fruits and vegetables demonstrate an importance for these factors [47,48,49,50,51]. Very few studies, however, have investigated these factors in relation specifically to vegetables. Vegetables differ from fruit in many respects; in their health benefits, usual consumption patterns, the determinants of consumption and the need for intervention [52]. To understand and increase vegetable-specific consumption, studies focusing solely on vegetables are required. Focus on the affective factors involved in vegetable consumption, furthermore, may provide more scope for intervention and change, while changing abilities and circumstances or the consequences of these may be more difficult.

This study aimed to investigate the affective factors associated with a high vegetable intake and a wide variety of vegetables consumed by older adults in Europe. Demographic predictors were also assessed as known predictors of vegetable or fruit and vegetable intakes in the older population [30,34,46,50,53,54,55,56,57,58]. However, our focus is on liking and attitudes, due to the higher potential for intervention and change. Analyses were conducted on data from three European countries—France, Italy and the UK. These countries represent different European cultures, cuisines and consumption patterns, particularly with respect to vegetables [25,59]. In Italy, for example, salad and raw vegetables are frequently consumed [25,60], while the traditional diet in the UK contains more cooked and more root vegetables [25,61].

## 2. Method

Data were collected as part of the VeggiEAT project, an EU funded project aiming to understand and increase vegetable intakes in adolescents and older adults from four European countries—Denmark, France, Italy and the United Kingdom. Data to assess self-reported consumption, liking for vegetables and attitudes towards consumption that may impact on vegetable consumption were assessed using questionnaires. The study was approved by the Research Ethics Committees of Bournemouth University, UK, the University of Firenze, Italy, Institut Paul Bocuse, France and the University of Copenhagen, Denmark, prior to commencement (bu id: 2657). Only the data on older individuals and only data from France (FR), Italy (IT) and the United Kingdom (UK) are analysed here. Insufficient data were collected from the older sample in Denmark to be included in these analyses.

### 2.1. Questionnaire

The questionnaire assessed various demographic characteristics, self-reported vegetable consumption, self-reported regular consumption of various vegetables, liking for various vegetables, and attitudes to food consumption, in that order.

Demographic characteristics: The demographic characteristics assessed were gender, age, country of residence, highest educational qualification (5 options, scored 1 (no formal qualifications)–5 (Postgraduate qualification)), current or recent level of full time employment (4 options, scored 1 (unemployed)–4 (professional/managerial employment)), and social affluence (4 questions, resulting in a score from 0 (low)–9 (high)).

Quantity of vegetable consumption: Vegetable consumption was assessed using a single item measure—‘*How many portions of vegetables do you consume in a usual day?*’, *response format: ‘none*’, ‘*1–2*’, *‘3–4*’, ‘*5 or more*’. A portion was defined as three tablespoons or 80 g of vegetables, for those who asked. Data were scored 0, 1.5, 3.5, and 5.5 respectively, to provide number of portions per day.

Variety of vegetables regularly consumed: Regular consumption of a variety of vegetables was assessed by asking for consumption of eleven vegetables of use in all four European countries [24]: ‘*broccoli*’, ‘*carrots’*, ‘*cauliflower’*, ‘*green beans’*, ‘*green salad*’, ‘*peas*’, ‘*spinach*’, ‘*sweetcorn*’, ‘*tomatoes*’, *‘courgettes*’, and ‘*beans*, *other than green beans*’. This question was included as part of a measure asking individuals to report their knowledge and frequency of consumption for all eleven vegetables, as used by Backstrom et al. [62]. Responses to the option ‘*I regularly eat this*’ were added to provide number of vegetables regularly consumed.

Quantity and variety of vegetables regularly consumed combined: Quantity and variety of vegetables regularly consumed were also combined, to provide a measure of consumption that would be most beneficial for health [23,24]. This combination was undertaken by reducing portions per day and number of vegetables regularly consumed to scores between 0 and 1 (by dividing number of portions per day by 5.5 and variety of vegetables consumed by 11), and multiplying these scores to provide a score from 0 to 1, where a higher score denotes a higher quantity of a variety of vegetables consumed. Reductions of the initial responses to a score between 0 and 1 ensured equivalence of quantity and variety variables.

Liking: Liking was assessed for each of the eleven vegetables above, on an individual basis using a nine-point scale ranging from ‘*I don*’*t like it at all*’ to *‘I neither like it nor don’t like it*’ to ‘*I like it a lot*’. Responses were scored −4 to 0 to +4 and the mean was calculated across all vegetables for analysis.

Attitudes: Attitudes towards food consumption were assessed using the Food Choice Questionnaire (FCQ) [63], the Restraint Scale of the Dutch Eating Behaviour Questionnaire (DEBQ-R) [64] and the Food Neophobia Scale (FNS) [65], and standard scoring procedures. On all three scales, higher scores denote higher motivation, dietary restraint, and food neophobia, respectively.

All questions were translated from English into relevant languages and back translated to ensure accurate translations. All questionnaires are frequently used to assess eating related attitudes and various studies demonstrate their applicability across countries and cultures [47,66,67,68,69,70]. The English version of the questionnaire can be found in Supplementary Materials I.

### 2.2. Questionnaire Administration

Questionnaires were administered in paper form either following a separate task assessing the sensory characteristics of several different pea and sweetcorn samples see [71], or following a separate task involving vegetable dish consumption in a cafeteria setting, or through the post as a study in it’s own right. For inclusion in either of the two tasks, individuals were required to be aged 65 years or over, able to come to the Institution undertaking the research, and able to fully understand and complete the consent and questionnaires. Participants were recruited by asking individuals who have previously participated in research, by contacting social groups for older people, and via advertisements in the local press. In both cases when tasks were undertaken, these were undertaken separately from completing the questionnaire, and are very unlikely to have had any impact on questionnaire responses. Researchers were available to answer questions if requested. For the postal study, individuals were known to research teams for having previously taken part in research, and were required to be aged 65 years or over. Contact with researchers remained possible via post, telephone and/or email. Multiple recruitment opportunities were used to enhance the variety of older adults completing the questionnaire. All individuals were living in the community (i.e., in their own homes or in a family home, and outside of residential care) at the time of assessment. All participants provided written informed consent. No other inclusion/exclusion criteria were used to enhance the generalisability of the study findings.

Questionnaires were administered until a sample size of at least 150 participants was gained per country, as required for the analyses we wished to conduct [72]. A minimum of 150 participants would allow the detection of an effect size of 0.15 in a regression analysis using 17 predictors, at a power of 0.80, for a significance level of 0.05.

### 2.3. Analysis

Questionnaires with 10% missing data or more were discarded. Where less than 10% data per respondent were missing, missing data were imputed using means for the country sample or mid-scale point values. Less than 2% of all data points were imputed, thus data imputation is likely to have had a minimal impact on our results while allowing use of more of the available data. Descriptive statistics and regression analyses were then run. Quantity of vegetables consumed, variety of vegetables regularly consumed, and quantity and variety combined were predicted using multiple linear regression. All analyses were conducted on quantity, variety and quantity × variety combined scores (0–1) for comparability. Outcomes were predicted using all demographic variables, where countries were ordered from North to South (UK, France, Italy), and all liking and attitude scales. A single country variable was used to retain as much power as possible in overall analyses. Analyses were conducted first on the overall sample (3 analyses—one investigating quantity; one investigating variety; one investigating quantity × variety). Due to significant impacts of country in these analyses, analyses were also conducted in each country separately (3 analyses per country, as above). In analyses on the Italian sample, consumption for reasons based on convenience were excluded from regression models due to high correlations with consumption for reasons based on price (*r* = 0.78, *p* < 0.01) and concerns over mutli-colinearity. Effects of price should thus be considered as effects of price/convenience in these analyses. No other concerns over multi-colinearity were found. Correlations between all predictor variables are provided in Supplementary Materials II. All analyses were conducted in SPSS (IBM, Armonk, NY, USA).

## 3. Results

### 3.1. Overall Sample

Complete data sets were gained from 497 individuals—187 individuals from France, 152 individuals from Italy, and 158 individuals from the UK. In the sample as a whole, mean quantity of vegetables consumed was low (mean = 2.1–2.7 portions/day) with a range from 0 to 5.5 portions; a mean of 6.1–6.7 different vegetables were consumed on a regular basis with a range from 0 to 11 vegetables; and scores for quantity and variety combined were low (mean = 0.19–0.36) with a range from the minimum possible to the maximum possible: 0–1. Descriptive details of the samples per country are provided in Table 1 and Table 2. Significant differences were found between countries in all demographic characteristics excepting the distribution of genders, and in measures of vegetable intake excepting the variety of vegetables consumed (smallest *F*(2,496) = 2.03, *p* = 0.13).

Quantity of vegetables consumed: Quantity scores were significantly predicted by the regression equation (*R* = 0.43, *R*^2^ = 0.19, adjusted *R*^2^ = 0.16, *F*(18,496) = 6.17, *p* < 0.01), where higher quantities of vegetable consumption were significantly associated with a higher age (β = 0.16, *p* < 0.01), a higher affluence score (β = 0.11, *p* = 0.03), living further North (β = −0.24, *p* < 0.01), a higher liking for vegetables (β = 0.16, *p* < 0.01) and a lower importance in consumption given to familiarity (β = −0.12, *p* = 0.04).

Variety of vegetables regularly consumed: Variety scores were significantly predicted by the regression equation (*R* = 0.54, *R*^2^ = 0.29, adjusted *R*^2^ = 0.26, *F*(18,496) = 10.63, *p* < 0.01), where greater variety of vegetable consumption was significantly associated with a higher liking for vegetables (β = 0.40, *p* < 0.01), a higher importance in consumption given to health benefits (β = 0.13, *p* = 0.02), and a lower importance in consumption given to familiarity (β = −0.11, *p* < 0.01). 

Quantity and variety of vegetables consumed combined: Combined quantity and variety scores were significantly predicted by the regression equation (*R* = 0.48, *R*^2^ = 0.23, adjusted *R*^2^ = 0.20, *F*(18,496) = 7.81, *p* < 0.01), where higher quantities of a varied vegetable consumption were significantly associated with a higher age (β = 0.11, *p* = 0.02), living further North (β = −0.20, *p* < 0.01), a higher liking for vegetables (β = 0.27, *p* < 0.01), a higher importance in consumption given to health benefits (β = 0.14, *p* = 0.02), and a lower importance in consumption given to familiarity (β = −0.17, *p* < 0.01). Results from the regression analyses on the whole sample are presented in Table 3.

### 3.2. French Sample

Results from the regression analyses on the French sample, including regression equations are presented in Table 4. Higher quantities of vegetable consumption were significantly associated with a higher age (β = 0.30, *p* < 0.01); a higher educational level (β = 0.23, *p* < 0.01), and a lower neophobia score (β = −0.15, *p* = 0.05). A greater variety of vegetable consumption was significantly associated with a lower age (β = −0.18, *p* < 0.01); a higher liking for vegetables (β = 0.36, *p* < 0.01), and a higher importance given to health benefits (β = 0.25, *p* < 0.01). Higher quantities of a varied vegetable consumption were significantly associated with a higher educational level (β = −0.20, *p* < 0.01); a higher liking for vegetables (β = 0.29, *p* < 0.01), and a lower neophobia score (β = −0.17, *p* = 0.02).

### 3.3. Italian Sample

Results from the regression analyses on the Italian sample, including regression equations are presented in Table 5. Higher quantities of vegetable consumption were significantly associated with a higher liking for vegetables (β = 0.36, *p* < 0.01). A greater variety of vegetable consumption was significantly associated with a higher liking for vegetables (β = 0.48, *p* < 0.01), and a higher importance in consumption given to price/convenience (β = 0.22, *p* = 0.03). Higher quantities of a varied vegetable consumption were significantly associated with a higher educational level (β = 0.19, *p* = 0.03), and a higher liking for vegetables (β = 0.51, *p* < 0.01).

### 3.4. UK Sample

Results from the regression analyses on the UK sample, including regression equations, are presented in Table 6. Higher quantities of vegetable consumption were significantly associated with a higher age (β = 0.19, *p* = 0.04), and a higher importance in consumption given to health benefits (β = 0.24, *p* = 0.05). A greater variety of vegetable consumption was significantly associated with a higher liking for vegetables (β = 0.31, *p* < 0.01), and a higher importance in consumption given to weight control (β = 0.19, *p* = 0.05). Higher quantities of a varied vegetable consumption were significantly associated with a higher age (β = 0.18, *p* = 0.04); a higher liking for vegetables (β = 0.19, *p* = 0.03), and a higher importance in consumption given to health benefits (β = 0.28, *p* = 0.02). 

A caution should be added to the findings on quantities of vegetable consumption in the UK sample, due to the lack of significance for the full regression model. This likely reflects a high contribution to the variance in quantity of vegetables consumed from variables that were not included in the regression model. Considering our *a-priori* interest in liking and attitudinal variables, we have continued to look at the individual predictors.

## 4. Discussion

Several key findings emerge from this study. Firstly, compared to World Health Organization guidelines (at least 160–240 g or 2–3 portions of vegetables/day), quantity, variety and quantity and variety of vegetables consumed combined were low, and several individuals reported either no vegetable consumption, or a regular consumption of no vegetables, or both. Our findings confirm those found in consumption databases [25], and extend these database findings to consider variety specifically. Both sets of findings demonstrate a need for intervention in this age group.

Secondly, quantity and variety of vegetables consumed were significantly associated with different demographic and attitudinal variables. Regarding demographic variables, higher quantities of vegetable consumption were associated with a higher age, and a higher affluence score, and higher quantities of a varied vegetable consumption were significantly associated with a higher age. Associations between vegetables consumption and age have previously been suggested [56,58], although reverse associations have also been found [53,73]. Together these findings suggest a relationship between vegetable consumption and age where vegetable consumption increases with age to a point and then drops off, presumably when age-related deteriorations in function and abilities and when chronic health conditions and disabilities begin to take effect [30,33,34,42,43,44,45,46,47]. Our findings most likely reflect the relatively young age of our older adults. Associations between vegetable consumption and affluence or socio-economic status have also previously been suggested [31,33,56,57,58], and associations between socio-economic status and healthy dietary and lifestyle habits are well known [28,31,50,53,56,58]. These associations have largely been associated with the monetary and practical costs of healthy dietary habits, and perceptions of fruits and vegetables as poor-value foods (in terms of energy provided per unit cost or unit effort) are reported [49,50,74]. These explanations may be over-simplifications to some degree [73], but monetary and practical costs may be particularly relevant to some older individuals [30,34,50].

Interestingly, however, affluence score was a predictor only of amount consumed, while variety of vegetables regularly consumed was not implicated. Other studies across Europe demonstrate a reverse association between affluence and intake to that found in the current study, where higher fruit and vegetable consumption has been reported in less affluent as opposed to more affluent individuals in Southern European countries, as a result of more traditional lifestyles [58]. This explanation may also explain our lack of association with variety. While money can essentially buy a lot of vegetables, eating a variety of vegetables may be associated both with being able to buy a variety of vegetables, but also with growing vegetables and with shopping for cheaper, seasonal vegetables, e.g., in markets. Growing vegetables and shopping for cheaper, seasonal vegetables are more likely to be the habits of less affluent as opposed to more affluent individuals. Variety of vegetables consumed thus may be unlikely to be associated overall with either a high or a low affluence.

Regarding liking and the attitudinal variables, greater quantity, variety and combined quantity and variety were associated with greater liking for vegetables and a lower concern for eating familiar foods, but a greater variety of vegetables consumed and a greater quantity of varied vegetable consumption were also associated with a greater concern for eating for health reasons.

Associations between vegetable consumption and liking are unsurprising. Repeated work demonstrates a role for liking in the consumption of vegetables throughout the lifespan [35,36,75,76], and previous work also demonstrates a role for liking in vegetable consumption and fruit and vegetable consumption in older consumers [34,47,48,49,50,77,78]. A role for liking in older consumers, while previously reported however, is important.

Associations between vegetable consumption and a lower concern for consuming familiar foods is interesting. This finding may reflect increased vegetable consumption in those willing to try different non-traditional preparation methods and dishes, e.g., stir-fries, or may demonstrate a necessary increase in willingness to consume unfamiliar dishes if a high vegetable consumption is to be attained. Pieniak et al. [69] report negative associations across Europe between traditional food consumption and concerns for a healthy diet, largely as a result of the high fat content of many traditional dishes [69]. Concern for health and a high consumption of vegetables thus may be associated with reduced preferences for traditional dishes and an increased willingness to consume unfamiliar, novel and unusual dishes. Alternatively, and/or additionally, associations between vegetable consumption and a willingness to try unfamiliar foods may be related to the bitter tastes often associated with vegetables [76,79], and may demonstrate the value of cooking methods and composite dishes where disliked tastes can be diluted or disguised [76,77,79]. In this case, unfamiliar dishes may include variations to familiar dishes as well as novel and unusual dishes. One other study in European older adults also finds an association between liking for fruit and vegetables and a willingness to try unusual foods [47].

The associations between consumption of a greater variety of vegetables (variety and quantity × variety combined) and greater concerns for eating for health reasons are also interesting. These findings suggest a particular role for health concerns in the consumption of a wide variety as opposed to a high quantity of vegetables. Consumption of a wide variety of vegetables will provide health benefits due to the different micronutrients found in different vegetables [11,12,13], and associations between health concerns and a variety of vegetables consumed suggest that consumers are aware of these associations. Previous studies report poor recognition of the different health benefits of different fruits and vegetables by younger consumers [74,80,81], but studies of or including older individuals suggest a better recognition of these benefits in older consumers [77,82]. When asked to specify the health conditions that benefit from fruit and vegetable consumption, older adults can spontaneously correctly identify a number of health conditions, including obesity, cardiovascular disease and some cancers [77], possibly as a result of a lifetime’s exposure to relevant public health information. Health knowledge and concern for consuming a healthy diet have also previously been associated with increased fruit and vegetable consumption in older adults [34,48,55,83].

Associations between vegetable consumption and liking, low concern for consuming familiar dishes and a high concern for consuming healthy dishes in the older age group are important findings. These findings suggest that interventions to improve vegetable consumption in this age group should focus on improving liking, increasing willingness to consume unfamiliar foods and dishes, and increasing knowledge of the health benefits of vegetables. Liking can be improved through repeated experience, increasing familiarity and the addition of ready-liked and familiar flavours to existing dishes. Various work demonstrates the value of these techniques for increasing likings for vegetables in different age groups [52] and work in older individuals demonstrates the value of the same techniques in these individuals for other foods, e.g., [84,85,86]. Willingness to consume unfamiliar foods can again be improved by repeated exposure and the addition of known and liked tastes and flavours, e.g., [87,88]. Opportunities for improving liking for vegetables through exposure to unfamiliar vegetables and dishes may also be possible through natural likings for flavours that are not yet known. The associations between vegetable consumption and willingness to consume unfamiliar dishes suggests that interventions need not involve familiar dishes. Liking and familiarity are often closely related [71], thus interventions that are based on familiar dishes, e.g., through the addition of vegetables to existing recipes, will likely be of benefit, but our findings also suggest that this is not necessary. Interventions that involve tasting novel fruit and vegetable products and involve different recipes and methods of cooking are typically well received [84,89,90], and can result in improvements in fruit and vegetable consumption [84,90]. These taste interventions may also be particularly useful for those of low socio-economic status, where new dishes tend to be avoided to reduce unaffordable waste [50].

Knowledge of the health benefits of vegetables and concern for consuming a healthy diet can be increased through educational and motivational campaigns [35], and various studies demonstrate the benefits of these types of intervention for increasing vegetable consumption in adults [52]. Health-based campaigns furthermore may be preferential both for increasing health benefits and for reducing socio-economic disparities, but we agree with others that liking and pleasure should be addressed alongside health campaigns [77]. In all our assessments, liking was a stronger predictor of intake than any other factor.

Higher quantities of vegetable consumption and a varied vegetable consumption were also associated with living further North. Higher consumption was found in the UK compared to France compared to Italy, but whether these effects are due to the country or due to the different samples studied in each country is unknown. Previous studies and consumption databases typically report a higher vegetable consumption in Southern as opposed to Northern European countries [25,33,57,58,60,73], and differences were found between the different samples for each country on several demographic variables, although genuine effects as a result of income/affluence from South to North have also been suggested [31]. Given the differences between samples, while the analyses within countries are likely to be accurate [47,67,68,69,70], direct comparisons between countries should only be conducted with caution.

Considering the analyses per country, in France, higher quantities of vegetable consumption were associated with a higher age, higher educational level, and a lower neophobia score; a greater variety of consumption was associated with a lower age, a higher liking for vegetables and a higher importance given to health benefits; and higher quantities of a varied vegetable consumption were associated with a higher educational level, a higher liking for vegetables, and a lower neophobia score. Effects due to increasing age, liking and an importance given to health benefits were found in the main analyses, and effects due to educational level and neophobia score reflect those found in the main analyses based on affluence score and the importance given to familiar foods respectively [58]. Effects of a lower age possibly reflect an importance given to familiar foods, as younger individuals (within the older age bracket (65 years plus)) tend to be more willing to consume less familiar foods, but an association between variety and younger age may also reflect the abilities required to consume a variety of vegetables [53].

In Italy, higher quantities of vegetable consumption were associated with a higher liking for vegetables, a greater variety of vegetable consumption was associated with a higher liking, and a higher importance given to price/convenience, and higher quantities of a varied vegetable consumption were associated with a higher educational level and a higher liking for vegetables. Effects of liking and educational level again reflect those in the main analyses. Effects between variety of vegetables consumed and an importance of price/convenience likely result from the traditional lifestyle in Italy. The majority of older individuals living in the community in Italy are likely to grow their own vegetables or shop for vegetables in local markets, to result in vegetable consumption that is cheaper and convenient [31,58,60]. In both these scenarios, the vegetables available will be seasonal, naturally resulting in a variety of vegetables consumed. Raw vegetables or large vegetable-based dishes such as soups and casseroles can also form cheap and easy meals, and these are staple foods of the Italian diet [60].

In the UK, higher quantities of vegetable consumption were associated with a higher age, a higher educational level, and a higher importance given to health benefits; a greater variety of consumption was associated with a higher liking for vegetables, and a higher importance given to weight control; and higher quantities of a varied vegetable consumption were associated with a higher age, a higher liking for vegetables, and a higher importance given to health benefits. Effects as a result of age, educational level, liking, and an importance of health benefits, again reflect those of the main analyses. Associations between variety of vegetables consumed and concerns regarding weight control may reflect a use of vegetables to control body weight specifically or advice for this behaviour as a result of the low energy density of vegetables [23,24,90]. These associations are thus also likely to be related to health concerns/benefits.

The consistency of many of the effects across countries is interesting and gives weight to these findings. While the patterns in the three countries are largely similar, some differences between countries may suggest the need for specific interventions in certain countries or population samples. Vegetable consumption in individuals living in communities served by local markets, for example, may benefit from interventions based on price, convenience and encouraging market shopping. Vegetable consumption in individuals with higher BMIs may benefit from interventions focussing on weight concerns. It is interesting furthermore, that the key differences between countries or between samples were found in variety of vegetables consumed, as opposed to in quantities consumed. These findings may suggest a further need for research specifically in this behaviour.

The strengths of the study include the consideration of vegetable consumption specifically as a food group of different consumption patterns, barriers to consumption and likely need for intervention compared to other foods [52], and the comparability of the results in three different European countries. The study is limited by the use of self-report questionnaires, and limited items to assess key outcomes. Particularly, quantity of vegetable consumption was assessed using a single question, variety was assessed using measures of the consumption of eleven pre-specified vegetables, while other vegetables may also be consumed, and no attempts were made to try and assess the accuracy of these measures using food diaries. Although self-report measures are commonly used in questionnaire studies of dietary behaviours, and brief measures have been reported as valid methods for measuring vegetable intake [91,92], these measures can be prone to inaccuracies and biases such as social desirability bias [93]. Our mean self-reported vegetable intakes (quantity) are notably higher than population averages [25,60] suggesting possible social desirability bias, but our sample may also be higher consumers of vegetables considering sufficient interest to participate in the study. While these measures may have resulted in slight inaccuracies in measures of vegetable intake, however, there is no reason to suspect any systematic bias in the associations between vegetable intake and food choice motives based on these measures. We also did not measure a number of lifestyle behaviours of potential impact on dietary intakes, such as living arrangements, social support and disabilities [30,33,34,46,55]. These were not measured to retain our focus on liking and attitudinal factors, but some of these factors may have increased the amount of variance predicted. Our findings are a result purely of the models considered. Thus, consideration of the liking and attitudinal factors in the absence of the demographic factors may also have changed the findings. Significant effects of socio-economic status, may be masking concerns in consumption due to price, for example. The study is also limited by small sample sizes in each of the individual countries and the particular details of these samples, e.g., data were only collected from individuals able to provide consent. Our results are thus limited to individuals who are relatively fit and able within the older population. The impact of this bias on associations between intakes and food choice motives remains unknown. The low comparability between country samples limits the cross-country conclusions that can be made. This variability between samples however, does not reduce the value of the findings from our main analyses, and in fact may increase the generalizability of these findings.

## 5. Conclusions

This study demonstrates a role for a higher liking and a lower concern for eating familiar foods in increased vegetable consumption (quantity, variety and quantity and variety combined), and a particular role for a greater concern for health benefits in the consumption of a greater variety of vegetables (variety and quantity and variety combined). These findings suggest that interventions to improve vegetable consumption in older adults should focus on improving liking, increasing willingness to consume unfamiliar foods and dishes, and increasing concern for health benefits.

## Figures and Tables

**Table 1:** Descriptive statistics (*n* and %, or mean and standard deviation) for demographic variables in all three countries (*n* = 497).

Characteristic		Total (*n* = 497)	France (*n* = 187)	Italy (*n* = 152)	UK (*n* = 158)
Gender	Male	160 (32%)	51 (27%)	57 (38%)	52 (33%)
	Female	337 (68%)	136 (73%)	95 (63%)	106 (67%)
**Age (years)**		**72.1 (6.7)**	**69.7 (5.3)**	**72.8 (7.0)**	**74.2 (7.0)**
**Highest instruction level**	**No qualifications**	**198 (40%)**	**78 (42%)**	**64 (42%)**	**56 (35%)**
	**School certificate**	**147 (30%)**	**33 (20%)**	**66 (43%)**	**43 (27%)**
	**College certificate**	**91 (18%)**	**33 (18%)**	**21 (14%)**	**37 (23%)**
	**University degree**	**47 (9%)**	**38 (20%)**	**1 (1%)**	**8 (5%)**
	**Professional qualification**	**14 (3%)**	**0**	**0**	**14 (9%)**
**Current/most recent employment**	**Unemployed**	**61 (12%)**	**31 (17%)**	**25 (16%)**	**5 (3%)**
	**Manual worker**	**62 (13%)**	**10 (5%)**	**37 (24%)**	**15 (10%)**
	**Non-manual worker**	**237 (48%)**	**105 (56%)**	**70 (46%)**	**62 (39%)**
	**Professional/management**	**137 (28%)**	**41 (22%)**	**20 (13%)**	**76 (48%)**
**Affluence (0–9)**		**4.5 (1.9)**	**4.5 (1.8)**	**4.0 (1.8)**	**5.1 (2.0)**

Significant differences between countries are emboldened.

**Table 2:** Descriptive statistics (mean, standard deviation) for quantity and variety of vegetable consumption and all attitudinal variables in all three countries (*n* = 497).

Characteristic	Total (*n* = 497)	France (*n* = 187)	Italy (*n* = 152)	UK (*n* = 158)
**Quantity of vegetables consumed (portions)**	**2.4 (0.7)**	**2.3 (0.6)**	**2.1 (0.5)**	**2.7 (0.8)**
**Quantity score (0–1)**	**0.4 (0.3)**	**0.4 (0.2)**	**0.3 (0.2)**	**0.6 (0.4)**
Different vegetables consumed regularly (number)	6.5 (2.9)	6.6 (2.8)	6.1 (3.2)	6.7 (2.6)
Variety score (0–1)	0.6 (0.3)	0.6 (0.2)	0.6 (0.3)	0.6 (0.2)
**Quantity x variety score (0–1)**	**0.3 (0.2)**	**0.24 (0.2)**	**0.19 (0.2)**	**0.35 (0.3)**
Liking (−4–+4)	2.5 (1.2)	2.8 (1.0)	2.4 (1.2)	2.4 (1.3)
FCQ—Mood (1–7)	4.8 (1.5)	4.5 (1.5)	5.7 (1.1)	4.2 (1.4)
FCQ—Sensory Appeal (1–7)	6.1 (1.0)	6.1 (1.0)	6.4 (0.8)	6.0 (1.0)
FCQ—Natural Content (1–7)	5.7 (1.3)	5.7 (1.5)	6.2 (0.9)	5.3 (1.3)
FCQ—Health (1–7)	6.0 (1.0)	6.1 (1.0)	5.9 (0.8)	5.8 (1.0)
FCQ—Convenience (1–7)	5.4 (1.2)	5.4 (1.2)	5.6 (1.1)	5.1 (1.3)
FCQ—Price (1–7)	5.3 (1.2)	5.3 (1.1)	5.5 (1.2)	5.0 (1.2)
FCQ—Weight Control (1–7)	5.2 (1.4)	5.3 (1.3)	5.5 (1.3)	4.7 (1.6)
FCQ—Familiarity (1–7)	5.1 (1.3)	5.4 (1.1)	5.7 (0.9)	4.4 (1.6)
FCQ—Ethical Concern (1–7)	4.9 (1.5)	4.9 (1.5)	5.6 (1.1)	4.4 (1.5)
DEBQ—R score (1–5)	2.7 (0.9)	2.9 (0.8)	2.5 (0.8)	2.7 (0.9)
Neophobia score (1–70)	31.7 (14.3)	32.7 (12.8)	28.5 (17.8)	33.4 (11.5)

**Table 3:** Results of the regression analyses using demographic characteristics and all liking and attitude variables to predict quantity, variety and combined quantity and variety scores in all participants (*n* = 497).

	Quantity		Variety		Combined Quantity and Variety	
	β	*p*	β	*p*	β	*p*
Gender	−0.17	0.71	<0.01	0.97	−0.02	0.58
Age	**0.16**	**<0.01**	−0.07	0.13	**0.11**	**0.02**
Instruction level	0.08	0.12	<0.01	0.98	0.06	0.22
Employment level	0.02	0.76	0.02	0.67	0.01	0.77
Affluence score	**0.11**	**0.03**	0.02	0.66	0.10	0.06
Country	**−0.24**	**<0.01**	−0.07	0.19	**−0.20**	**<0.01**
Liking	**0.16**	**<0.01**	**0.40**	**<0.01**	**0.27**	**<0.01**
FCQ—Mood	0.05	0.36	−0.06	0.23	0.04	0.41
FCQ—Sensory Appeal	0.06	0.25	0.06	0.19	0.08	0.08
FCQ—Natural Content	0.02	0.80	0.05	0.34	0.02	0.76
FCQ—Health	0.08	0.19	**0.13**	**0.02**	**0.14**	**0.02**
FCQ—Convenience	−0.02	0.80	0.06	0.30	−0.01	0.89
FCQ—Price	0.01	0.83	−0.04	0.47	−0.01	0.90
FCQ—Weight Control	−0.11	0.07	0.04	0.42	−0.06	0.29
FCQ—Familiarity	**−0.12**	**0.04**	**−0.11**	**0.04**	**−0.17**	**<0.01**
FCQ—Ethical Concern	0.05	0.34	0.05	0.36	0.05	0.35
DEBQ—Restraint	0.01	0.76	−0.01	0.84	−0.01	0.92
Neophobia score	−0.03	0.53	−0.05	0.23	−0.03	0.48

Significant predictors are emboldened.

**Table 4:** Results of the regression analyses using demographic characteristics and all liking and attitude variables to predict quantity, variety and combined quantity and variety scores in French participants (*n* = 187). Regression equations: Quantity: *R* = 0.46, *R*^2^ = 0.21, adjusted *R*^2^ = 0.13, *F*(17,186) = 2.60, *p* < 0.01; Variety: *R* = 0.67, *R*^2^ = 0.45, adjusted *R*^2^ = 0.40, *F*(17,186) = 8.27, *p* < 0.01; Combined quantity and variety: *R* = 0.46, *R*^2^ = 0.21, adjusted *R*^2^ = 0.13, *F*(17,186) = 2.60, *p* < 0.01.

	Quantity		Variety		Combined Quantity and Variety	
	β	*p*	β	*p*	β	*p*
Gender	−0.07	0.34	0.04	0.52	−0.05	0.44
Age	**0.30**	**<0.01**	**−0.18**	**<0.01**	0.10	0.19
Instruction level	**0.23**	**<0.01**	−0.01	0.91	**0.20**	**0.01**
Employment level	<0.01	0.99	−0.02	0.72	−0.07	0.36
Affluence score	0.08	0.35	−0.03	0.70	0.06	0.47
Liking	0.09	0.26	**0.36**	**<0.01**	**0.29**	**<0.01**
FCQ—Mood	0.09	0.28	−0.06	0.39	0.07	0.36
FCQ—Sensory Appeal	<0.01	0.98	0.13	0.09	0.09	0.28
FCQ—Natural Content	0.12	0.22	0.01	0.95	0.07	0.46
FCQ—Health	−0.06	0.55	**0.25**	**<0.01**	0.08	0.41
FCQ—Convenience	0.07	0.51	0.04	0.66	0.09	0.32
FCQ—Price	−0.08	0.34	−0.06	0.40	−0.11	0.18
FCQ—Weight Control	−0.09	0.35	−0.04	0.60	−0.09	0.33
FCQ—Familiarity	0.07	0.51	0.01	0.90	0.07	0.48
FCQ—Ethical Concern	0.10	0.23	0.06	0.39	0.08	0.34
DEBQ—Restraint	0.09	0.28	0.03	0.67	0.07	0.34
Neophobia score	**−0.15**	**0.05**	−0.12	0.06	**−0.17**	**0.02**

Significant predictors are emboldened.

**Table 5:** Results of the regression analyses using demographic characteristics and all liking and attitude variables to predict quantity, variety and combined quantity and variety scores in Italian participants (*n* = 152). Regression equations: Quantity: *R* = 0.46, *R*^2^ = 0.21, adjusted *R*^2^ = 0.12, *F*(16,151) = 2.28, *p* = 0.01; Variety: *R* = 0.64, *R*^2^ = 0.41, adjusted *R*^2^ = 0.34, *F*(16,151) = 5.78, *p* < 0.01; Combined quantity and variety: *R* = 0.59, *R*^2^ = 0.34, adjusted *R*^2^ = 0.27, *F*(16,151) = 4.41, *p* < 0.01.

	Quantity		Variety		Combined Quantity and Variety	
	β	*p*	β	*p*	β	*p*
Gender	−0.08	0.35	−0.04	0.57	−0.07	0.40
Age	<0.01	0.99	−0.07	0.40	−0.04	0.68
Instruction level	0.17	0.09	0.16	0.06	**0.19**	**0.03**
Employment level	0.11	0.29	−0.10	0.26	0.05	0.62
Affluence score	0.04	0.67	0.04	0.66	0.03	0.74
Liking	**0.36**	**<0.01**	**0.48**	**<0.01**	**0.51**	**<0.01**
FCQ—Mood	−0.04	0.67	−0.10	0.29	−0.06	0.56
FCQ—Sensory Appeal	0.05	0.60	−0.07	0.37	−0.04	0.61
FCQ—Natural Content	0.04	0.73	0.12	0.21	0.13	0.22
FCQ—Health	−0.09	0.50	0.10	0.40	0.06	0.62
FCQ—Convenience	-	-	-	-	-	-
FCQ—Price/Convenience	−0.09	0.41	**0.22**	**0.03**	0.07	0.52
FCQ—Weight Control	−0.11	0.41	−0.11	0.34	−0.19	0.13
FCQ—Familiarity	−0.05	0.63	−0.10	0.24	−0.09	0.28
FCQ—Ethical Concern	0.01	0.92	−0.05	0.64	−0.07	0.50
DEBQ—Restraint	0.11	0.22	−0.02	0.77	0.04	0.66
Neophobia score	0.04	0.61	−0.01	0.88	0.08	0.33

Significant predictors are emboldened.

**Table 6:** Results of the regression analyses using demographic characteristics and all liking and attitude variables to predict quantity, variety and combined quantity and variety scores in UK participants (*n* = 158). Regression equations: Quantity: *R* = 0.36, *R*^2^ = 0.13, adjusted *R*^2^ = 0.03, *F*(17,157) = 1.26, *p* = 0.23; Variety: *R* = 0.49, *R*^2^ = 0.24, adjusted *R*^2^ = 0.15, *F*(17,157) = 2.64, *p* < 0.01; *R* = 0.43, *R*^2^ = 0.18, adjusted *R*^2^ = 0.08, *F*(17,157) = 1.85, *p* = 0.03.

	Quantity		Variety		Combined Quantity and Variety	
	β	*p*	β	*p*	β	*p*
Gender	0.01	0.92	−0.04	0.65	−0.02	0.79
Age	**0.19**	**0.04**	0.12	0.16	**0.18**	**0.04**
Instruction level	0.02	0.84	−0.07	0.43	−0.01	0.89
Employment level	−0.04	0.64	0.01	0.90	−0.02	0.79
Affluence score	0.12	0.19	0.07	0.41	0.11	0.24
Liking	0.13	0.15	**0.31**	**<0.01**	**0.19**	**0.03**
FCQ—Mood	0.05	0.60	0.03	0.73	0.07	0.48
FCQ—Sensory Appeal	0.07	0.49	0.14	0.14	0.10	0.30
FCQ—Natural Content	−0.09	0.40	−0.02	0.88	−0.10	0.34
FCQ—Health	**0.24**	**0.05**	0.14	0.24	**0.28**	**0.02**
FCQ—Convenience	−0.18	0.20	−0.17	0.18	−0.21	0.11
FCQ—Price	0.04	0.72	−0.09	0.36	0.03	0.76
FCQ—Weight Control	−0.09	0.38	**0.19**	**0.05**	−0.02	0.84
FCQ—Familiarity	−0.11	0.36	−0.20	0.09	−0.19	0.12
FCQ—Ethical Concern	0.02	0.77	0.01	0.96	0.04	0.66
DEBQ—Restraint	−0.02	0.87	0.02	0.80	−0.01	0.95
Neophobia score	0.01	0.96	0.15	0.11	0.05	0.63

Significant predictors are emboldened.

## Supplementary Materials

The following are available online at www.mdpi.com/2072-6643/9/9/923/s1.
