# Supplementary material for: Consumption of a High Quantity and a Wide Variety of Vegetables Are Predicted by Different Food Choice Motives in Older Adults from France, Italy and the UK
Source: Nutrients. 2017 Aug 23;9(9):923. doi: 10.3390/nu9090923 (PMC5622683; doi:10.3390/nu9090923)
Supplement: Supplementary file 1 [file nutrients-09-00923-s001.zip › nutrients-209349-supplementary-final check/nutrients-209349 - Supplementary Materials I.pdf]

Code

veggiEAT

Please complete the following questionnaire as accurately as possible. Please answer all questions.

1, Gender

2. Age

3. Thinking about the house you live in at the moment, do you own it or rent it (If you have a mortgage, tick you own it?)
□ own it
□ rent it

4. Do you have a car, van or truck at home?
□ yes, one car or van
□ yes, more than one car or van
□ no, we don't own a car or van

5. How many computers do you own? (Do not include playstations or other computers that can only be used for games)
□ none
□ one
□ two
□ more than two

6. During the past 12 months, how many times did you travel away on holiday?
□ not at all
□ once
□ twice
□ more than twice

7. What is your highest educational qualification?
□ no formal qualifications
□ school leaving certificate / exams
□ college certificates / exams
□ University degree
□ professional qualification beyond University degree

8. What was your most recent level of employment, prior to retirement?
□ unemployed
□ manual worker
□ non-manual worker
□ professional or management

9. How many portions of vegetables (not including potatoes) do you eat in a usual day?
□ none
□ 1-2
□ 3-4
□ 5 or more

10. Please rate your familiarity with the vegetables in the list. Tick the appropriate phrase for each product

	I do not recognize this	I recognize this, but I have not tasted it	I have tasted, but I do not eat this	I occasionally eat this	I regularly eat this
broccoli					
carrots					
cauliflower					
green beans					
green salad					
peas					
spinach					
sweet corn					
tomatoes					
courgettes					
beans, other than green beans					

11. How much do you like the vegetables in the list? Tick the appropriate rating for each vegetable

	Dislike extremely				Neither like or dislike				Like extremely
	1	2	3	4	5	6	7	8	9
broccoli									
carrots									
cauliflower									
green beans									
green salad									
peas									
spinach									
sweet corn									
tomatoes									
courgettes									
beans, other than green beans									

12. For each question, please place a tick in the column which best applies to you.

	never	seldom	some-times	often	very often
If you have put on weight, do you eat less than you usually do?					
Do you try to eat less at mealtimes than you would like to eat?					
How often do you refuse food or drink offered because you are concerned about how much you weigh?					
Do you watch exactly what you eat?					
Do you deliberately eat foods that are slimming?					
When you have eaten too much, do you eat less than usual on the following days?					
Do you deliberately eat less in order not to become heavier?					
How often do you try not to eat between meals because you are watching your weight?					
How often in the evening do you try not to eat because you are watching your weight?					
Do you think about your weight before deciding how much to eat?					

13. I am constantly sampling new and different foods

strongly disagree strongly agree

☐ ☐ ☐ ☐ ☐ ☐ ☐

14. I don’t trust new foods

strongly disagree strongly agree

☐ ☐ ☐ ☐ ☐ ☐ ☐

15. If I don’t know what is in a food, I won’t try it

strongly disagree strongly agree

☐ ☐ ☐ ☐ ☐ ☐ ☐

16. I like foods from different countries

strongly disagree strongly agree

☐ ☐ ☐ ☐ ☐ ☐ ☐

17. Food from countries different to mine looks too weird to eat

strongly disagree ☐ ☐ ☐ ☐ ☐ ☐ ☐ strongly agree

18. At social gatherings I will try a new food

strongly disagree ☐ ☐ ☐ ☐ ☐ ☐ ☐ strongly agree

19. I am afraid to eat things I have never eaten before

strongly disagree ☐ ☐ ☐ ☐ ☐ ☐ ☐ strongly agree

20. I am very particular about the foods I will eat

strongly disagree ☐ ☐ ☐ ☐ ☐ ☐ ☐ strongly agree

21. I will eat almost anything

strongly disagree ☐ ☐ ☐ ☐ ☐ ☐ ☐ strongly agree

22. I like going to restaurants that serve foods from different countries

strongly disagree ☐ ☐ ☐ ☐ ☐ ☐ ☐ strongly agree

23. For each question, please place a tick in the column which best applies to you.

It is important to me that the food that I eat on a typical day:

	I strongly disagree	I disagree	I disagree a little	I neither agree nor disagree	I agree a little	I agree	I strongly agree
Helps me cope with stress							
Helps me to cope with life							
Helps me relax							
Keeps me awake/alert							
Cheers me up							

24. For each question, please place a tick in the column which best applies to you.

It is important to me that the food that I eat on a typical day:

	strongly disagree			neutral			strongly agree
Makes me feel good							
Smells nice							
Looks nice							
Has a pleasant texture							
Tastes good							
Contains no additives							
Contains natural ingredients							
Contains no artificial ingredients							
Contains a lot of vitamins and minerals							
Keeps me healthy							
Is nutritious							
Is high in protein							
Is good for me							
Is high in fibre and roughage							
Is easy to prepare							
Can be cooked very simply							
Takes no time to prepare							

25. For each question, please place a tick in the column which best applies to you.

It is important to me that the food that I eat on a typical day:

	strongly disagree			neutral			strongly agree
Can be bought in shops close to where I live or work							
Is easily available in shops and supermarkets							
Is not expensive							
Is cheap							
Is good value for money							
Is low in calories							
Helps me control my weight							
Is low in fat							
Is what I usually eat							
Is familiar							
Is like the food I ate when I was a child							
Comes from countries I approve of politically							
Has the country of origin clearly marked							
Is packaged in an environmentally friendly way							

Many thanks for your help with this questionnaire, it is much appreciated.
