# Supplementary material for: Consumption of a High Quantity and a Wide Variety of Vegetables Are Predicted by Different Food Choice Motives in Older Adults from France, Italy and the UK
Source: Nutrients. 2017 Aug 23;9(9):923. doi: 10.3390/nu9090923 (PMC5622683; doi:10.3390/nu9090923)
Supplement: Supplementary file 1 [file nutrients-09-00923-s001.zip › nutrients-209349-supplementary-final check/nutrients-209349 - Supplementary Materials II.pdf]

Supplementary Materials II

Table S1. Pearson correlation co-efficients between all dependent variables for the overall sample (*n* = 497).

	**Age**	**Education**	**Employment**	**Affluence**	**Liking**	**FCQ—Mood**	**FCQ—SA**	**FCQ—NC**	**FCQ—Health**	**FCQ—C**	**FCQ—Price**	**FCQ—WC**	**FCQ—F**	**FCQ—EC**	**DEBQ—R**	**Neo—phobia**
Gender	−0.047	0.167 **	0.143 **	0.222 **	−0.120 **	0.041	0.054	−0.044	−0.079	−0.069	−0.013	−0.091*	−0.015	−0.014	−0.091*	−0.019
Age		−0.154 **	−0.069	−0.240 **	−0.208 **	0.065	0.023	−0.113 *	−0.096 *	0.042	−0.023	−0.051	0.040	−0.083	−0.217**	0.152**
Education			0.403 **	0.373 **	0.076	−0.085	0.050	0.026	0.033	−0.179**	−0.068	−0.058	−0.226**	−0.037	0.156**	−0.040
Employment				0.294 **	0.052	−0.174 **	0.012	−0.006	−0.017	−0.138 **	−0.064	−0.049	−0.193**	−0.072	0.114*	0.096*
Affluence					0.101 *	−0.212 **	0.017	0.033	−0.026	−0.232 **	−0.262 **	−0.130 **	−0.235**	−0.056	0.111*	−0.024
Liking						−0.099 *	−0.033	0.204 **	0.217 **	0.086	0.084	0.212 **	0.051	0.132**	0.198**	−0.147**
FCQ—Mood							0.340 **	0.355 **	0.324 **	0.297 **	0.180 **	0.333 **	0.296**	0.336**	0.057	−0.117**
FCQ—Sensory appeal								0.368 **	0.404 **	0.215 **	0.170 **	0.190 **	0.251**	0.259**	0.066	0.025
FCQ—Natural content									0.532 **	0.200 **	0.142 **	0.467 **	0.279**	0.471**	0.227**	−0.065
FCQ—Health										0.447 **	0.310 **	0.521 **	0.372**	0.358**	0.269**	−0.056
FCQ—Convenience											0.598 **	0.432 **	0.558**	0.327**	0.021	0.112*
FCQ—Price												0.428 **	0.363**	0.265**	0.064	0.037
FCQ—Weight control													0.415**	0.429**	0.348**	−0.025
FCQ—Familiarity														0.405**	0.028	0.112*
FCQ–ethical concern															0.082	−0.051
DEBQ–R																−0.038

* significant $p < 0.05$, ** significant $p < 0.01$.

Table S2. Pearson correlation co-efficients between all dependent variables for the French sample (n = 187).

	**Age**	**Education**	**Employment**	**Affluence**	**Liking**	**FCQ—Mood**	**FCQ—SA**	**FCQ—NC**	**FCQ—Health**	**FCQ—C**	**FCQ—Price**	**FCQ—WC**	**FCQ–F**	**FCQ–EC**	**DEBQ–R**	**Neo—phobia**
Gender	−0.046	0.251 **	0.182 *	0.248 **	−0.105	0.124	0.207 **	−0.043	−0.048	−0.116	−0.095	−0.103	−0.059	−0.009	0.042	−0.032
Age		−0.252 **	−0.197 **	−0.343 **	−0.231 **	0.066	−0.077	−0.239 **	−0.196 **	0.132	0.161*	−0.127	0.048	−0.067	−0.274 **	0.308 **
Education			0.359 **	0.458 **	0.063	−0.006	0.202 **	0.157 *	0.066	−0.190 **	−0.192**	−0.047	−0.224 **	0.071	0.260**	−0.177 *
Employment				0.193 **	0.141	−0.005	0.213 **	0.185 *	0.125	0.016	0.047	0.071	−0.029	0.093	0.209 **	−0.078
Affluence					0.221 **	−0.102	0.253 **	0.255 **	0.143	−0.048	−0.213 **	0.050	−0.093	0.048	0.246 **	−0.133
Liking						−0.063	0.099	0.292 **	0.266 **	0.099	−0.120	0.247 **	0.089	0.156 *	0.242 **	−0.227 **
FCQ—Mood							0.299 **	0.239 **	0.304 **	0.311 **	0.117	0.248 **	0.174 *	0.140	0.144 *	0.052
FCQ—Sensory appeal								0.451 **	0.443 **	0.230 **	0.124	0.210 **	0.385 **	0.295 **	0.170 *	0.004
FCQ—Natural content									0.517 **	0.275 **	0.099	0.426 **	0.262 **	0.464 **	0.335 **	−0.055
FCQ—Health										0.480 **	0.260 **	0.525 **	0.415 **	0.399 **	0.277 **	−0.180 *
FCQ—Convenience											0.488 **	0.507 **	0.568 **	0.344 **	0.043	0.073
FCQ—Price												0.424 **	0.435 **	0.255 **	−0.013	0.155 *
FCQ—Weight control													0.462 **	0.362 **	0.307 **	−0.023
FCQ—Familiarity														0.437 **	0.050	0.152 *
FCQ–ethical concern															0.151 *	−0.029
DEBQ—R																−0.103

* significant $p < 0.05$, ** significant $p < 0.01$.

Table S3. Pearson correlation co-efficients between all dependent variables for the Italian sample (n = 152).

	Age	Education	Employment	Affluence	Liking	FCQ—Mood	FCQ—SA	FCQ—NC	FCQ—Health	FCQ—C	FCQ—Price	FCQ—WC	FCQ—F	FCQ—EC	DEBQ—R	Neo—phobia
Gender	−0.192 *	0.026	0.195 *	0.253 **	−0.124	−0.164 *	−0.010	−0.054	−0.062	0.039	0.061	−0.095	−0.076	−0.032	−0.118	0.084
Age		−0.078	−0.190 *	−0.201 *	−0.198 *	−0.027	0.209 **	−0.003	−0.063	−0.263 **	−0.273 **	−0.030	0.150	−0.140	−0.148	0.076
Education			0.511 **	0.380 **	−0.046	−0.210 **	−0.007	0.120	0.005	0.031	0.018	0.048	−0.125	0.126	−0.049	0.249**
Employment				0.443 **	−0.063	−0.127	−0.022	0.017	−0.127	−0.073	−0.028	−0.022	−0.263 **	0.058	−0.010	0.251**
Affluence					−0.150	−0.207 *	−0.093	−0.039	−0.147	−0.239 **	−0.265 **	−0.196 *	−0.326 **	−0.093	0.043	0.138
Liking						0.002	−0.130	0.206 *	0.237 **	0.349 **	0.410 **	0.363 **	0.190 *	0.241 **	0.217 **	−0.127
FCQ—Mood							0.225 **	0.347 **	0.527 **	0.245 **	0.208 *	0.393 **	0.277 **	0.391 **	0.221 **	−0.166 *
FCQ–Sensory appeal								0.166 *	0.222 **	0.089	0.103	0.125	0.112	0.174 *	0.128	0.219 **
FCQ—Natural content									0.614 **	0.270 **	0.233 **	0.632 **	0.092	0.555 **	0.265 **	0.076
FCQ—Health										0.535 **	0.382 **	0.663 **	0.402 **	0.656 **	0.282 **	0.063
FCQ—Convenience											0.783 **	0.490 **	0.452 **	0.544 **	0.109	0.021
FCQ—Price												0.508 **	0.311 **	0.525 **	0.141	−0.024
FCQ—Weight control													0.250 **	0.615 **	0.428 **	0.064
FCQ—Familiarity														0.183 *	0.076	−0.066
FCQ—ethical concern															0.166 *	0.026
DEBQ—R																−0.042

* significant $p < 0.05$, ** significant $p < 0.01$.

Table S4. Pearson correlation co−efficients between all dependent variables for the UK sample (n = 158).

	**Age**	**Education**	**Employment**	**Affluence**	**Liking**	**FCQ—Mood**	**FCQ—SA**	**FCQ—NC**	**FCQ—Health**	**FCQ—C**	**FCQ—Price**	**FCQ—WC**	**FCQ—F**	**FCQ—EC**	**DEBQ—R**	**Neo—phobia**
Gender	0.030	0.220 **	0.091	0.207 **	−0.093	0.029	−0.092	−0.077	−0.104	−0.126	−0.009	−0.094	0.036	−0.057	−0.140	−0.125
Age		−0.143	0.020	−0.311 **	−0.102	0.139	−0.033	−0.033	0.052	0.282 **	0.100	0.081	0.169 *	−0.028	−0.157 *	0.164 *
Education			0.347 **	0.238 **	0.129	0.108	0.002	−0.035	−0.004	−0.221 **	0.074	−0.036	−0.173 *	−0.054	0.094	−0.235 **
Employment				0.112	0.120	−0.094	−0.071	−0.012	−0.077	−0.265 **	−0.096	0.026	0.002	−0.053	0.070	−0.048
Affluence					0.224 **	−0.155	−0.063	−0.011	−0.108	−0.337 **	−0.244 **	−0.124	−0.152	0.054	−0.003	−0.215 **
Liking						−0.156 *	−0.056	0.163 *	0.118	−0.135	−0.026	0.067	−0.079	0.093	0.078	−0.153
FCQ—Mood							0.350 **	0.318 **	0.372 **	0.250 **	0.119	0.274 **	0.182 *	0.247 **	0.117	−0.097
FCQ—Sensory appeal								0.305 **	0.510 **	0.238 **	0.222 **	0.151	0.136	0.160 *	0.010	−0.085
FCQ—Natural content									0.566 **	−0.006	0.047	0.357 **	0.197*	0.305 **	0.226 **	−0.118
FCQ—Health										0.363 **	0.317 **	0.450 **	0.367 **	0.209 **	0.222 **	−0.067
FCQ—Convenience											0.541 **	0.285 **	0.601 **	0.116	−0.031	0.356 **
FCQ—Price												0.327 **	0.298 **	0.024	0.114	0.054
FCQ—Weight control													0.351 **	0.292 **	0.405 **	−0.052
FCQ—Familiarity														0.299 **	0.051	0.436 **
FCQ—ethical concern															0.111	−0.007
DEBQ—R																−0.066

* significant $p < 0.05$, ** significant $p < 0.01$.
